# ­­­Mechanosensitivity of aged muscle stem cells

**DOI:** 10.1002/jor.23797

**Published:** 2017-12-18

**Authors:** Heleen E. Boers, Mohammad Haroon, Fabien Le Grand, Astrid D. Bakker, Jenneke Klein‐Nulend, Richard T. Jaspers

**Affiliations:** ^1^ Laboratory for Myology Faculty of Behavioural and Movement Sciences Vrije Universiteit Amsterdam Amsterdam Movement Sciences De Boelelaan 1108 1081 HZ Amsterdam The Netherlands; ^2^ Department of Oral Cell Biology Academic Centre for Dentistry Amsterdam University of Amsterdam and Vrije Universiteit Amsterdam Amsterdam Movement Sciences Amsterdam The Netherlands; ^3^ Sorbonne Universités UPMC Univ Paris 06 INSERM UMRS974 CNRS FRE3617 Center for Research in Myology 75013 Paris France

**Keywords:** muscle stem cell, satellite cell, aging, mechanosensitivity, muscle regeneration, mechanotransduction

## Abstract

During aging, skeletal muscle tissue progressively declines in mass, strength, and regenerative capacity. Decreased muscle stem cell (MuSC) number and impaired function might underlie the aging‐related muscle wasting and impaired regenerative capacity. As yet, the search for factors that regulate MuSC fate and function has revealed several biochemical factors within the MuSC niche that may be responsible for the decline in MuSC regenerative capacity. This decline cannot be explained by environmental factors solely, as the MuSC potential to regenerate muscle tissue is not reversed by changing the biochemical MuSC niche composition. Here we discuss the likeliness that during physical exercise, MuSCs within their niche are subjected to mechanical loads, in particular pressure and shear stress, as well as associated deformations. We postulate that these physical cues are involved in the activation and differentiation of MuSCs as these cells contain several transmembrane sensor proteins that have been shown to be mechanosensitive in other cell types, that is, endothelial cells and osteoprogenitors. We will specifically address age‐related changes in mechanosensing in MuSCs and their niche. Insight in the physical cues applied to the MuSCs in vivo, and how these cues affect MuSC fate and function, helps to develop new therapeutic interventions to counterbalance age‐related muscle loss. This requires an approach combining two‐ and three‐dimensional live cell imaging of MuSCs within contracting muscle tissue, mathematical finite element modeling, and cell biology. © 2017 The Authors. *Journal of Orthopaedic Research*® Published by Wiley Periodicals, Inc. on behalf of the Orthopaedic Research Society. J Orthop Res 36:632–641, 2018.

The age‐related loss of muscle mass and muscle strength, or sarcopenia, is associated with impaired physical function, increased risk of falls, fractures, and dependency on major health care concern for the aged individual. Hence it is very important to prevent loss of muscle mass at advanced age. The causes of muscle dysfunction during aging are subject of intense scrutiny, but the cellular mechanisms underlying this dysfunction remain elusive. Presumably sarcopenia is caused by loss of myofibers and subsequent replacement with fibrotic tissue,^1^ myonuclear apoptosis and myofiber atrophy.^2^

Prevention of myofiber loss and myofiber atrophy relies on adequate regenerative capacity of the muscle stem cells (MuSCs), also referred to as satellite cells, and on the potential of myofibers to synthesize proteins. In injured muscle, activated MuSCs, repopulate the injured segments along the myofibers.^3^ In response to mechanical overload by exercise or stretching, MuSCs are also activated and proliferate to fuse with the host myofiber. In the old muscle, accretion of myonuclei in myofiber by proliferation and fusion of MuSCs is required to replace apoptotic myonuclei within aged myofibers.^4^ This accretion is needed to increase the pool of myonuclei, which subsequently enhances the rate of protein synthesis and counterbalances muscle atrophy.^5^ Several studies report a progressive decrement of MuSC population with age^6^ and impaired function of MuSC in aged muscles,^7^ but the mechanisms underlying the age‐related decline in muscle regenerative capacity are still not fully understood.

MuSCs are located in a unique niche enclosed by a myofiber plasma membrane (sarcolemma) and lamina densa of the basal lamina (Fig. 1). Physical exercise‐induced mechanical overloading of myofibers activates quiescent MuSCs resulting in a population of transiently amplifying myoblasts expressing the muscle regulatory factors MyoD and Myf5.^8^ Then most myoblasts permanently exit the cell cycle and fuse to form new myofiber segments and regenerate muscle tissue, while a sub‐population of MuSCs undergoes self‐renewal and re‐populates the stem cell niche. During this regeneration process, biochemical signals from the local microenvironment, such as insulin‐like growth factor (IGF‐1) and mechano growth factor (MGF), myostatin, transforming growth factor‐β (TGFβ), interleukin‐6 (IL‐6), and tumor necrosis factor‐α (TNFα) are involved in MuSC activation and/or differentiation, while Wnt signaling pathways instruct cycling of MuSCs and control myogenic fate choice.^3^ MuSC activation and fate decision are clearly affected by paracrine biochemical cues from neighboring host myofibers, fibroblasts, and adipocytes, or by endocrine biochemical cues from the circulation. Strong support for a systemic basis of the age‐related impairment of MuSC function has been derived from heterochronic parabiosis studies in aged and young mice. Sharing the circulation systems of old and young mice normalizes the regenerative capacity of aged muscle in response to injury, suggesting that the absence of particular serum factors and the aged muscle composition, are critically determining MuSC function.^9^

**Figure 1:**
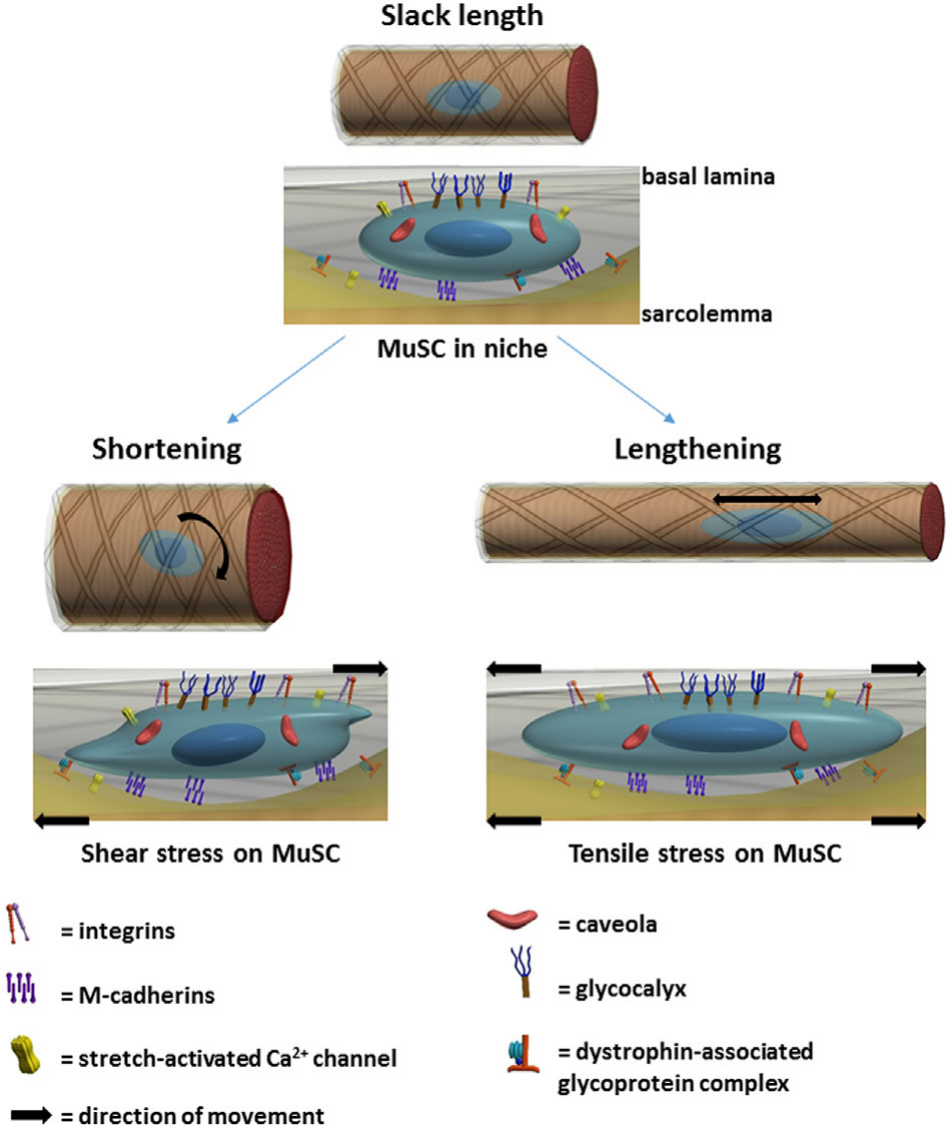
Schematic showing effects of stretch‐shortening on MuSC orientation and deformation. The myofiber is ensheathed by the sarcolemma (yellow) and the basal lamina (BL) surrounded by a collagen fiber reinforced matrix (gray sheath with black crossing lines). Top figure is a segment of an unstrained myofiber. Below the myofiber is an enlarged lateral view of the MuSC in its niche, illustrating the well‐known transmembrane proteins, anchoring the MuSC to sarcolemma and BL. While the myofiber is unstrained, MuSCs in their niches are also in an unloaded state. Upon myofiber shortening, the sarcolemma will move relative to the BL. Since, the MuSC is anchored to both sheaths, it is likely to twist from a longitudinal orientation towards a more radial orientation (direction of arrow). As the myofiber is shortening, the sarcolemma and BL are likely moving at varying speeds, the relative movement of the sheaths will impose a shear force onto the MuSC, with subsequent cellular deformation. Myofiber lengthening will stretch both the sarcolemma and the BL (direction of arrow), which may induce a tensile stress on the MuSC.

The heterochronic parabiosis studies clearly show a positive effect of supplying favorable circulating factors from young mice on regeneration of aged muscle, however recent evidence suggests that aged MuSCs, outside their aged environment in well‐defined conditions, are still impaired in function and fate decision.^10^ Aged mouse MuSCs grafted into young mouse muscle maintain a compromised regenerative capacity upon injury of the young muscle.^10^ Thus the MuSC potential to regenerate muscle tissue cannot be simply reversed by changing the environmental conditions in the MuSC niche, suggesting that old MuSCs are intrinsically different from young. DNA damage and disturbed signalling cascades might be responsible for the lack of intrinsic regenerative capacity of the aged MuSC.^11^ The aged niche conditions of MuSCs are known to cause epigenetic changes in the regulation of gene expression, which strongly determine MuSC fate and function.^12^ However, it is also possible that in addition to these changes, or as a result of these changes, aged MuSCs do not perceive mechanical loads appropriately. Environmental stiffness has recently been shown to determine proliferative capacity of aged MuSC, as aged MuSCs cultured on a compliant substrate show increased proliferation rate, compared to cells cultured on a stiff substrate.^10^

Muscles are subjected to stretch‐shortening during daily life movements, and even more during physical exercise. Muscle regeneration after myofiber injury is enhanced by post injury physical exercise.^13^ In general, physical exercise is considered a safe way to maintain or enhance muscle mass. In theory, it is an attractive therapy to counterbalance sarcopenia, but its efficacy to prevent loss of muscle mass likely declines with age.^6^, ^14^ This suggests that there are changes in the way mechanical stimuli are perceived by myofibers with aging, or in the way muscles process the stimuli to increase mass.^15^ Upon stretch‐shortening contractions, the extracellular matrix (ECM, i.e., endomysium) is subjected to mechanical loads and deformations such as tensile and shear strains,^16^, ^17^ as well as compression. Since the MuSCs is enclosed by ECM, the MuSC in its niche is likely also subjected to these forces. In vitro studies have shown that stretch of MuSC induces production of factors which are directly involved in activation, proliferation and self‐renewal of these cells.^18^ In vivo studies on MuSCs are scarce, but smooth muscle cells in vivo are highly sensitive to mechanical loads, influencing proliferation, differentiation, migration and apoptosis.^19^ Mechanical cues on MuSCs may be one of the determinants for MuSC activation, proliferation, and differentiation.

In this perspective we hypothesize that aging‐related dysfunction of MuSCs is due, at least in part, to impaired MuSC mechanosensitivity, in addition to alterations in extrinsic and/or intrinsic biochemical factors. We postulate that: (i) MuSCs are highly mechanosensitive, (ii) during physical exercise MuSC are subjected to mechanical loads within their niche and (iii) during aging MuSC mechanosensitivity is impaired, resulting in reduced regenerative capacity upon injury. The impaired mechanosensitivity of aged MuSC would contribute to an attenuated accretion of myonuclei to the host muscle fiber to compensate for myonuclear apoptosis, causing an increase in myonuclear domain size.

A thorough understanding of the mechanism of age‐related muscle loss will provide a starting point for the development of new therapeutic interventions and/or tissue engineering strategies. Currently it is unknown whether exercise therapy can help to prevent sarcopenia in the elderly. Changing the frequency, intensity, and/or exercise conditions (aerobic, anaerobic) might increase the regenerative capacity of aged muscle.

## INTERACTION OF MUSCLE STEM CELL WITH ITS NICHE COMPONENTS

MuSCs are sandwiched in their niches, between the lipid bilayer of the myofiber (i.e., sarcolemma) and ECM, consisting of the basal lamina containing collagen IV, laminin and fibronectin.^20^ The MuSC membrane contains several transmembrane complexes via which the cell is anchored to the niche components. At the interface with the host myofiber, the MuSC is connected via cadherins, which are calcium‐dependent cell‐cell adhesion proteins that span the cell membrane. Inside the cell, these receptors are connected to the cytoskeleton via several catenin proteins, thus creating a continuous connection of the matrix outside with the inside of the cell. A cell‐matrix connection of MuSCs to basal lamina components is established via integrins and the dystrophin‐associated glycoprotein complex (DGC), a complex containing the molecules dystrophin and dystroglycans.^21^ Dystrophin connects DGCs to actin filaments inside the cell, while dystroglycans link the complex to laminin outside the cell. Integrins are heterodimer transmembrane proteins linked to the cytoskeleton via focal adhesion proteins.

In healthy adult muscle tissue, MuSCs are retained in a quiescent state until activation by mechanical loading and/or damage to myofibers. The activation of MuSCs depends on a complex interaction between physical and biochemical niche conditions (see for an overview^3^). The physical properties of the niche are determined by ECM proteins such as fibronectin, laminin, collagen, and their cross‐linking (see for review^20^). The membrane‐enclosed niche contains a multitude of soluble biochemical factors derived from the circulation, or secreted by other cells in the ECM surrounding myofibers, such as fibroblasts, endothelial cells and/or adipocytes.^3^ Biochemical factors in the niche, as well as physical niche properties, may change acutely during stretch‐shortening contraction, or adapt to physical exercise in the long term. The acute effects of mechanical loading on the niche properties result in activation of the MuSCs to proliferate, self‐renew, and repopulate the niche, or to differentiate and fuse with the myofiber.^8^ The interaction between niche conditions and MuSC properties such as cell stiffness, adherence, transcriptional regulators, and epigenetics, will determine MuSC fate, i.e., self‐renewal or differentiation. Taken together this process of MuSC activation in response to mechanical loading will increase the number of myonuclei in the myofiber, supporting hypertrophy and regeneration of myofibers after injury.

During aging both biochemical factors and physical properties of the niche change, which are characterized by elevated levels of cytokines and growth factors as well as increased ECM stiffness. Partly due to these changes, an adequate MuSC response to mechanical loading and/or injury fails to appear.^11^ It remains to be elucidated how a mechanical interaction between MuSC and niche affects the MuSC fate and function. The contribution of the MuSC itself to an adequate loading response, compared to the contribution of its niche components, will be virtually impossible to study as these will be indistinguishable in vivo.

## MECHANOSENSING COMPLEXES OF MUSCLE STEM CELLS

For MuSCs to respond to mechanical loading and biophysical niche properties, they must have the ability to sense these mechanical cues. This ability is an important quality of stem cells in general, and crucial in determining mesenchymal stem cell fate.^22^ In addition, for stem cell migration to the injured site, adhesion capacity and dynamic cytoskeleton arrangement are required. Cell migration is crucial in the regeneration of damage myofibers, highlighting the function of mechanosensors in MuSC.

### Transmembrane Complexes in MuSC

Several transmembrane complexes, such as integrins, cadherins, DGCs and syndecans, have been identified in the MuSC to connect the cell to surrounding membranes or proteins (see for a review^3^) (Fig. 1).

Integrins consist of an α‐ and β‐unit, which adhere to ECM proteins outside the MuSC. The focal adhesions inside the cell consist of multiple proteins, for example, talin, paxillin, vinculin, and α‐actinin. The adhesion capacity of the cell‐matrix connection via integrins depends on the maturation of focal adhesions, a progressive clustering of integrins and abovementioned proteins. In endothelial cells, blood flow‐induced shear stress on the luminal side is transferred from the glycocalyx via the cytoskeleton to integrin clusters on the basal side of the cells. This results in the maturation of focal adhesions on the basal side of the cell, increasing the adhesion to the underlying structure.^23^ Within the MuSC niche there is no fluid flow like in arteries and capillaries, however it is conceivable that the endomysium enclosing the MuSCs is subject to shear stress^17^, ^24^ and the maturation of focal adhesion could be an important process in adhesion or migration of MuSC along the myofiber.

The most abundant integrin subunits in muscle and MuSCs are α7 and β1. These two subunits form a heterodimer that binds to laminin in the ECM. Importantly, it has recently been shown that integrin‐β1 is an essential transmembrane protein necessary for MuSC maintenance of quiescence and self‐renewal following injury.^25^ In differentiating MuSCs, integrin‐β1 regulates the formation of a protein complex important for fusion and the subsequent sarcomere assembly.^26^ Interestingly, blocking of integrin β1 in myotubes leads to a decline in NO production upon stretching.^27^ NO radicals initiate molecular programs in MuSCs that lead to muscle growth and regeneration, and they are up‐regulated by exercise and injury.^18^ The α‐subunit has a major impact on muscle function as well, as enhanced expression of integrin α7 in dystrophic mice restores the viability of muscle tissue.^28^ Detrimental effects of integrin blocking on cell viability and the muscle response to mechanical loading have been reported, indicating that the transmission of force via these receptors is likely important for MuSC fate and function.

DGC is another receptor binding to laminin, which was first thought to be present only in myofibers. Recently, it has been shown that DGC is also expressed in MuSCs.^21^ The expression of DGC‐encoding genes in MuSCs is even higher than in myotubes. Proliferation of MuSCs in DGC‐deficient mouse is diminished in vitro and muscle tissue from DGC‐deficient mouse displays impaired regeneration. The effect of DGC‐deficiency on mechanoresponsiveness of MuSCs is however unknown. It is plausible that the DGC is important in MuSC sensing of mechanical cues, as the complex is excellently situated, being connected to laminin outside the cell and to the actin cytoskeleton inside the cells.

At the interface between the MuSC and the myofiber, M‐cadherins form cell‐cell connections. Inside the MuSC, M‐cadherin is linked to actin filaments via α‐and β‐catenin, which in other cell types has been shown to regulate cell shape and adhesive capacity. M‐cadherin is likely involved in overload‐induced hypertrophy, as the percentage of myofibers with MuSCs expressing both M‐cadherin and β‐catenin increases after mechanical overload in rat plantaris muscle.^29^ It remains to be determined whether the increase in M‐cadherin and β‐catenin expression is induced by the myofiber expressing more M‐cadherin receptors, by the MuSC responding to the load applied and/or by cellular deformation. In addition, the relation between increased M‐cadherin expression by MuSCs and myofiber hypertrophy is unclear. As M‐cadherin connects the MuSC to the myofiber, this transmembrane complex may be important in the sensing of myofiber stretch‐shortening.

Stretch activated Calcium (Ca^2+^) channels embedded in the membrane of MuSCs are important regulators of intracellular calcium levels. Cell deformation via shear stress or direct pressure on MuSCs result in opening of these channels, but this has not been reported so far. The stretching of myoblasts, leads to the influx of Ca^2+^‐ions via the opening of these channels,^18^ demonstrating the intrinsic capacity of these membrane molecules to serve as mechanosensing complexes in MuSCs.

**Figure 2:**
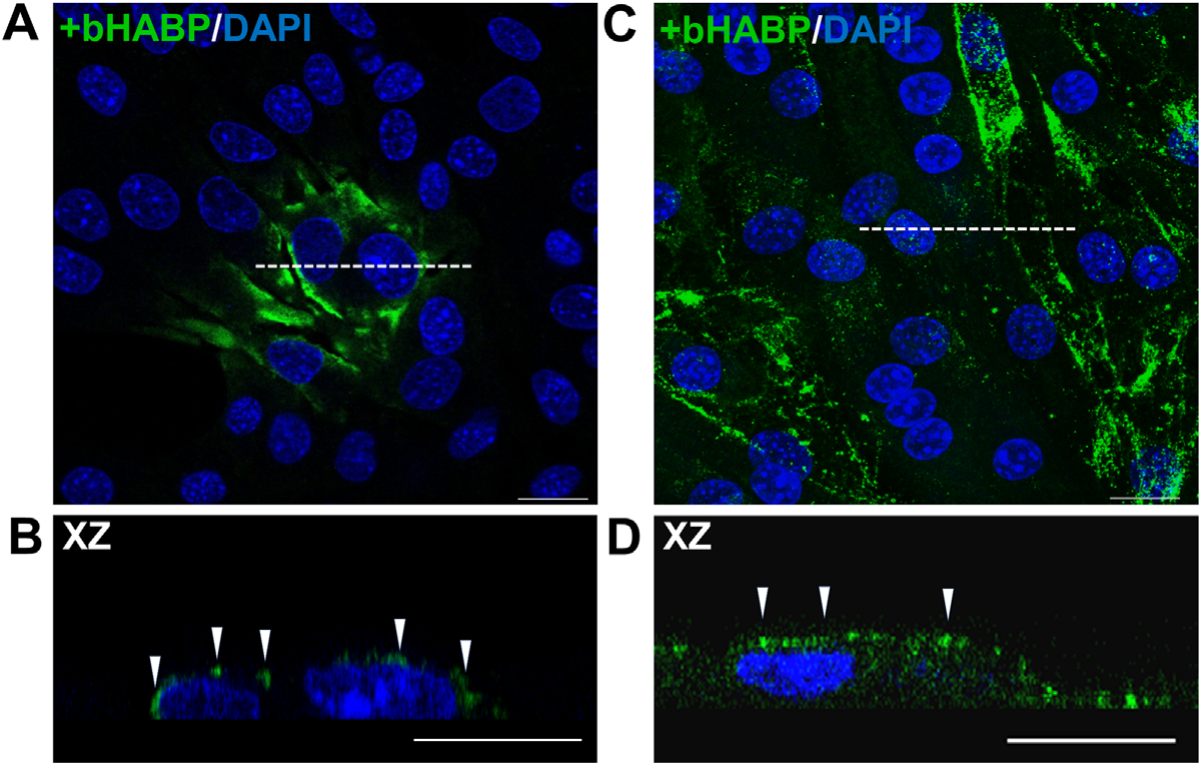
Glycocalyx expression in C2C12 myoblasts and differentiated myotubes. (A,B) Immunofluorescent images of myoblasts cultured for 3 days and stained against hyaluronic acid (component of glycocalyx) by biotinylated hyaluronic acid binding protein (bHABP, green) and counterstained with DAPI (blue). (A) Top view image of cells after 3 days of culture show that only few myoblasts express hyaluronic acid (green). (B) Cross‐sectional image of myoblasts, as indicated by the white dotted line in figure A, shows that hyaluronic acid matrix is on top of the myoblast (white arrows). (C) C2C12 myotubes cultured for 7 days show abundant expression of hyaluronic acid. (D) Cross‐sectional image of myotubes shows hyaluronic acid at the myotube membrane (white arrows). Scale bars indicate 20 μM. These results confirm the presence of a glycocalyx on top of myotubes, whereas after 3 days of culture only few myoblasts show glycocalyx expression. For myotubes, enzymatic removal of hyaluronic acid matrix abolished the pulsating fluid shear stress induced NO production,^30^ indicating its importance for mechanosensing. Since most myoblasts do not seem to be covered by hyaluronic acids, mechanosensing might be different from that of cells with a well‐developed glycocalyx.

The glycocalyx, including the core transmembrane proteins (i.e., syndecans and glypicans), is another transmembrane structure that might be important for sensing different types of mechanical loading (Fig. 2). The glycocalyx surrounding myotubes has been shown to be important for mechanosensing of shear stress.^30^ Preliminary data of our lab show that myoblasts have lower levels of glycocalyx in vitro. It remains to be determined whether this is also the case in vivo, but the presence of a glycocalyx adds yet another potential mechanosensing complex to the already extensive toolbox of MuSCs.

### MuSC Cytoskeleton Determines its Stiffness and Shape

The mechanical properties of MuSCs have not been studied in detail, but in general cells control their own stiffness by regulating the number of cytoskeletal filaments, their cross‐linking and interactions with actomyosin.^31^ The latter allows cytoskeletal traction, cell movement, and adjustment of cell shape to the morphology and physical properties of the environment. Mechanical properties of mesenchymal stem cells have been extensively examined and are strongly related to the ability of these cells to differentiate into tissue specific cells (adipocytes, osteoblasts, chondrocytes, skeletal muscle cells and smooth muscle cells (see for a review^32^). The regulation of actin filament content within a cell is a dynamic process, depending on interactions of the MuSC with niche structures and mechanical loads applied onto the cell. Integrins regulate these physical properties through regulating the activation of members of the Ras homolog gene family, such as RhoA. This enhances actin polymerization, regulates cell shape, motility and polarity. In endothelial cells, expression of RhoA and subsequent actin polymerization is determined by the mechanical loading of the cells (shear, compression, and/or stretch).^32^ The level of F‐actin in cells determines the resistance to mechanical loading.

To the best of our knowledge, stiffness of MuSCs has not been qualified or quantified yet. Given the ability of other stem cells to regulate their cytoskeletal composition in response to mechanical loading, this could be relevant for MuSC as well. Two different shapes of MuSCs have recently been distinguished, that is, round MuSCs with blebs on the surface, or flat MuSCs with a spread, lamellipodial based membrane.^33^ The migration speed of round, blebbing cells is higher, compared to that of flat, lamellipodial based cells. The formation of blebs is highly dynamic, and it likely depends on actin polymerization. The stiffness of these cells is however unknown, and it remains to be determined whether these two cell shapes are present within muscle in vivo as well.

Taken together, the presence of transmembrane receptors and the composition of the cytoskeleton of MuSCs suggest that MuSCs possess all the tools to enable them to sense mechanical stimuli and to give an adequate response to mechanical loading.

## FORCE TRANSMISSION FROM MUSCLE TO STEM CELL

To understand how mechanical cues can affect MuSC function and fate decision, a detailed description of force transmission from muscle to stem cell is required. Body movements are accomplished by force exertion of myofibers on the skeleton while shortening or being stretched by antagonist muscle activation. A myofiber consists of myofibrils arranged in parallel, constituting sarcomeres linked in series. Myofibers are embedded in a honeycomb ECM which forms a tube of connective tissue around the myofiber (Fig. 1). The perimysium surrounds bundles of myofibers, and the epimysium surrounds the whole muscle, connecting the muscle to the bones. Forces exerted by the sarcomeres are transmitted to the inter‐myofibrillar cytoskeleton and via trans‐sarcolemmal proteins onto the ECM. Sarcolemmal force is transmitted to the tendon at the myotendinous junction or along the length of the myofiber via the ECM (i.e., myofascial force transmission).^16^ Finite element modeling has shown that serial sarcomere strain within a muscle is not uniform, resulting in shear stresses onto the ECM.^16^, ^24^ Forces from the myofiber and movement of the ECM are likely transmitted to the MuSC, as the MuSC is anchored to both of these structures (Fig. 1). During stretch‐shortening of myofibers, MuSCs might be subjected to tensile stress, pressure and shear stress. Below we will discuss how the forces from the myofiber and the relative movement of connective tissue result in MuSC force subjection.

### Tensile Stress

During movement, myofibers undergo substantial length changes, either passively or while actively exerting force. Stretching of myofibers opens stretch activated calcium channels (SACs) and activates integrin signaling pathways.^18^, ^34^ It has recently been shown that end‐to‐end tensile myofiber strain causes elongation of myonuclei.^35^ This raises the question whether MuSCs are also lengthened when the host myofiber is stretched. As the MuSC is connected to both the sarcolemma and basal lamina, it is conceivable that the MuSC will be subjected to tensile stress and undergo tensile strain deformation (Fig. 1). Mechanical stretch of myoblasts increases micro‐RNA expression of proliferation related genes,^36^ NO production and HGF expression,^18^ indicating that these cells are mechanoresponsive to tensile stress. To the best of our knowledge, no evidence exists that indeed MuSCs are subjected to tensile stress and undergo strain deformation during physical exercise.

### Pressure

The volume of myofibers remains constant during contractions, which is an important feature already described in the 17th century by Jan Swammerdam. The constancy of muscle cell volume has major implications for the ECM and the pressure applied to the MuSC. Upon shortening, serial arranged filaments slide along each other causing a radial outward force. When myofibers shorten simultaneously, MuSCs sandwiched between adjacent myofibers, are compressed. After severe injury, cyclic compression in mouse tibialis anterior muscle increases its regenerative capacity and decreases fibrosis.^37^ During physical exercise in humans, however, concentric contractions fail to increase the number of MuSCs in the m. vastus lateralis, whereas eccentric contractions increase MuSC number.^38^ In addition, electrical stimulation of myotubes in vitro has been reported to enhance sarcomere assembly.^39^ Under compression, C2C12 myoblasts change their shape and increase their contact surface with the substrate.^40^ Such a change in cell shape will cause membrane deformations which will likely activate SACs, integrins, DGCs, and cadherins. The in vivo effects of cyclic compression on muscle regeneration indicate that MuSCs are sensitive to pressure. It remains to be determined though what type of physical exercise is most effective, as mechanical loading of muscle by concentric contraction solely does not increase the number of MuSCs, whereas exercise including eccentric contractions does.

Besides direct pressure on the MuSC, myofiber shortening might lead to local variations in interstitial fluid pressure, as is observed in passively lengthened myofibers.^41^ Variation in fluid pressure results in a displacement of interstitial fluid. In bone, fluid displacement within the canaliculi results in the application of shear stress on the osteocytes. Shear stress in bone is critical for osteocytes function and bone adaptation to mechanical loading. Although the ECM of muscle differs substantially from the canaliculi in which osteocytes protrude, fluid displacement also occurs in the ECM of muscle fibers due to pressure, and may induce shear loading onto MuSCs.

### Shear Stress

Shear loading of the ECM in muscle in vivo occurs, as myofibers possess lateral linkages to the ECM via which sarcomere force is likely transmitted.^42^ Adjacent myofibers may contract at different speeds, due to variation in myosin‐heavy chain isoform expression, which causes local strain distributions. Local differences in stiffness of the sarcolemma and ECM, such as neurovascular tracts, are likely contributing to shear loading of the ECM surrounding the myofibers.^17^ Moreover, as the volume of the myofiber does not change upon shortening or stretching, the orientation of collagen fibrils change from a predominantly longitudinal alignment with the myofiber to a more radial orientation. MuSCs are anchored to these fibrils and follow the deformation of the ECM (Fig. 1). When the MuSC would not be anchored to the sarcolemma and the basal lamina, the membrane and connective tissue would slide along the MuSC. However, given the transmembrane complexes, MuSCs likely follow the change in orientation of the sarcolemma and ECM upon myofiber stretch and shortening. Due to the local differences in stiffness in the ECM, the sarcolemma and ECM are not moving exactly simultaneously which makes it conceivable that MuSCs are frequently subjected to shear stress.

**Figure 3:**
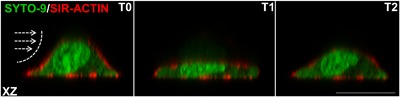
Live cell imaging of C2C12 myoblast cell deformation in response to static laminar fluid shear stress. Myoblasts were seeded on glass coverslips coated with poly‐l‐lysin and cultured for 1 day. Cells were stained against nucleic acid (Syto‐9, green) and F‐actin (Sir‐actin, red) for 3 h. The coverslip was mounted in a parallel‐plate flow chamber and a static laminar fluid shear stress (FSS) (0.97 Pa) was generated by pumping 7 ml culture medium (DMEM with 2% FBS, penicillin, streptomycin, and fungizone) through the flow chamber for 5 min as described previously.^50^ Confocal XZ (cross‐sectional) images were taken before subjecting cells to FSS (T0) and about 7 s and 30 s after the start of FSS (at T1 and T2, respectively). Immediately, after application of FSS, the myoblast transiently collapsed (T1), however at T2 the myoblast had regained its original shape. The direction of FSS is indicated by white arrows. Scale bar is 20 μM. These data underline the substantial impact of FSS on morphology of the cell, including the cell membrane, cytoskeleton, and nucleus.

Other cell types, such as endothelial cells and osteocytes, are mechanically loaded by fluid displacement in vivo, inducing a shear stress onto these cells.^43^, ^44^ By mimicking such loads in vitro, it has been shown that osteocytes are highly responsive to pulsating fluid flow as this type of loading stimulates NO production and expression of genes associated with osteocyte‐to‐osteoclast and osteocyte‐to‐osteoblast communication.^45^ Shear stress induced by fluid displacement differs significantly from the way a MuSC would be subjected to shear stress following different changes in orientation and displacement of sarcolemma and ECM. However, the pulsating fluid flow model used to study the effect of fluid shear stress on endothelial and bone cells in vitro has been applied to myotubes. These myotubes showed several responses which are similar to those of osteocytes.^30^ Our group, a collaboration between bone and muscle departments, has recently subjected MuSCs to shear stress using the fluid flow model. Preliminary data of live cell confocal imaging in combination with novel nuclear and cytoskeletal actin probes allows visualization of how fluid shear stress induces substantial deformation of the myoblast, its cytoskeleton, and the nucleus. Acutely upon the application of fluid shear stress, the cell collapses and retains its initial shape shortly after the start of the load while the flow was continuous (Fig. 3). These data show that MuSCs respond to shear stress.

The studies available on mechanotransduction of MuSCs in vitro are restricted to the use of tensile strain as mechanical stimulus.^18^ Myotubes, however, that were subjected to shear stress by our group using fluid flow, showed an increase in expression of growth factors and cytokines,^30^ involved in the regulation of myofiber size, as well as in MuSC activation and differentiation. It is hitherto unknown whether the response of MuSCs to fluid shear stress in vitro exactly recapitulates the activation of signaling pathways, or the expression of regulatory factors seen upon mechanical loading of muscle in vivo.

### Assessment of in Situ Mechanical Loading of MuSCs

To date very little is known about the mechanical loads and deformations MuSCs are subjected to in vivo. Experimental invasive approaches to get access to MuSCs in their natural surroundings requires interference with the intramuscular connective tissue, which likely affects the mechanical conditions of the MuSC. An approach which allows to study the effects of muscle contraction, as well as those of shortening and lengthening on MuSC deformation is by studying ex vivo myofiber bundles using multiphoton imaging of MuSCs in transgenic mice models with site‐specific recombinase and inducible gene expression system. This approach allows live‐cell imaging of MuSCs and their membranes within their niches while being subjected to mechanical loads associated with active and passive stretch‐shortening of the myofibers. Myofiber shortening or lengthening, combined with MuSC deformation and MuSC stiffness can be used as parameters in a finite element model to determine what forces are exerted onto MuSCs. The effects of these forces on aged MuSC function and fate decision can be studied in vitro, using fluid flow chambers, flexible‐bottomed culture plates or by pulling on transmembrane proteins, such as integrins.^31^

All types of loading, that is, tensile stress, pressure and shear stress, are likely exerted onto MuSCs in vivo and might be important determinants of MuSC function and fate in response to mechanical loading, in addition to the biochemical factors in the niche. In vivo, however, forces exerted onto MuSCs will be a combination of different types of loading, that is, stretch, pressure and shear stress, resulting in complex MuSC deformations.

## EFFECT OF AGING ON MECHANOSENSING

During aging, the properties of muscle tissue, MuSCs and their niche gradually change. In general the wear and tear of skeletal muscle results in fibrosis and accumulation of adipose tissue, which likely increases ECM stiffness. The adhesion of MuSCs to their niches is crucial in determining MuSC function and fate.^11^, ^25^, ^46^ In addition, mechanosensing of the aging MuSC may become dysfunctional due to intrinsic changes of the MuSC by alterations in expression levels of transmembrane and/or cytoskeletal proteins.

### Biophysical Properties of the Aging Niche

Stiffness‐determining components of the MuSC niche change during aging. The basal lamina thickens, which increases the stiffness of this sheath.^47^ The concentration of collagen IV increases significantly in slow twitch muscles, whereas laminin levels become elevated in fast twitch muscle in aged rats.^48^ The concentration of the extracellular matrix protein fibronectin is decreased in the aged MuSC niche. The loss of fibronectin is related to a lower regenerative capacity of muscle in old mice.^46^ These alterations in biophysical niche properties change the stiffness of the MuSC micro‐environment, which affects forces encountered by the MuSC.

### Transmembrane Proteins in Aged MuSC

In aged mouse MuSC, several substantial changes in integrin levels have been reported. Integrin‐α7β1 expression levels in aged MuSCs are decreased, and disorganized patterns of integrins are observed compared to young MuSCs.^25^, ^33^ In addition to changes in integrin subunits in aged MuSCs, spatial distributions of focal adhesion proteins, paxillin and vinculin, are disorganized.^25^ Moreover, aged muscle shows reduced expression of fibronectin within its ECM, which results in loss of integrin adherence to the ECM, impaired integrin signaling and reduced MuSC selfrenewal.^35^ Inhibition of integrin‐β1 in young MuSC is associated with impaired regeneration of young myofibers ex vivo, and results in reduced adhesion capacity in vitro.^24^, ^46^ In contrast, activation of integrin signaling in aged muscle improve the regenerative capacity of MuSCs.^24^ These results suggest that the aging related loss in integrin and fibronectin as well as the disorganized spatial distribution of integrin‐β1 have detrimental effects on the mechanical adherence of the MuSC to its niche and limits its regenerative capacity. Given the importance of integrins in mechanosensation, the impaired integrin signaling in aged MuSCs indicates that mechanosensing of these cells in old age is diminished. Apart from changes in integrin content and their spatial distribution, the expression and localization of other molecules involved in mechanosensing of aging MuSC remains an under‐exposed subject.

### Cytoskeleton Stiffness and Cell Shape of the Aged MuSC

Apart from possible alterations in the mechanosensors of aged MuSC, the shape and stiffness of these cells might be changed as well. As the activation of integrin‐dependent RhoA signaling regulates actin polymerization in other cell types,^32^ a change in integrin content in MuSCs is expected to indirectly alter the stiffness of these cells. This implicates that aged MuSCs subjected to external forces will have a diminished potential to alter their own rigidity and adhesion, which could be a reason for their reduced regenerative potential.

Preliminary observations of aged MuSCs cultured on Matrigel in our lab, show some typical differences in MuSC shape. Aged MuSC show less cell spreading and more cells have a round morphology. This could be related to the level of actin filaments inside the cell, and adherence of the cell to the matrix proteins. The stiffness of MuSCs has not been assessed thus far. It would be interesting to compare the stiffness of young and aged MuSC.

### Cause and Effect of Dysfunctional Mechanosensing of Aged MuSCs

In vivo the effects of changes to biophysical niche properties on muscle function will be difficult to distinguish from changes in intrinsic MuSC mechanisms for mechanosensing, but should be separated to uncover the mechanisms underlying the impaired regenerative capacity in aging muscle. To determine whether the mechanosensitivity of aged MuSCs is intrinsically impaired, and if so, by which mechanisms, a detailed quantification of expression of transmembrane complexes is required as well as their spatial localization. This requires RNA sequencing and/or quantitative immunohistochemistry of, for example integrins, cadherins, and focal adhesion proteins. In addition, signaling molecules directly downstream of the mechanosensors, for example NO production, calcium influx and focal adhesion formation, should be studied in aged MuSCs outside their aged niche. The acute response of these molecules to mechanical loading precedes the cellular pathways for activation of gene transcription and differentiation and is likely not directly affected by epigenetic changes to the chromatin. In case particular components of the mechanosensitive system are affected, restoration of their expression should reveal the contribution of impaired MuSC mechanosensitivity to the overall MuSC regenerative capacity.

In addition, a more detailed characterization of the epigenetic basis of impaired MuSC function is required to develop targeted therapeutical approaches to reprogram these epigenetic modifications. Such reprogramming and subsequent testing of its impact on mechanosensitivity and MuSC function will provide indications how epigenetic modifications and impaired mechanosensitivity are related. Within the proposed in vitro and ex vivo studies, culture conditions are well defined and standardized which allows to obtain insight in the effects of particular physical and biochemical niche conditions. However, to assess how effects of modulation in gene expression of MuSCs and changes in their mechanosensitivity apply to the MuSCs within an aged niche, in vivo studies are warranted. These studies should not only study MuSC function and regenerative capacity but should also assess whether prevention or restoration of aging‐related dysfunction in MuSC mechanosensitivity slows muscle fiber loss, atrophy and fibrosis, and maintains force generating capacity of the aged muscle.

## CONCLUSION AND FUTURE PERSPECTIVES

The MuSC in the MuSC niche in connection to extra‐muscular connective tissue will likely be subjected to mechanical loads during physical exercise. The presence of several mechanosensory complexes within the MuSC membrane combined with evidence of mechanoresponsiveness of MuSCs indicates that mechanical loads and subsequent cellular deformations are important co‐determinants of MuSC activation and differentiation. During aging, expression and localization of transmembrane complexes in MuSCs are disturbed, which likely affects MuSC function and fate decision, resulting in impaired regeneration and response to mechanical loading. Yet, studies on factors that regulate MuSC fate in both young and aged muscle have mostly focused on the MuSC (biochemical) environment. Here we propose a more detailed investigation of the biophysical aspects of both the MuSC and its niche.

It remains to be established how MuSCs are loaded in vivo and how the cells themselves, their membrane, as well as the mechanosensors are deformed. This requires live cell confocal imaging of MuSCs within their niche inside the muscle or in small bundles of myofibers which can be stimulated for contraction and which are allowed to undergo stretch‐shortening cycles while being imaged. Therefore, 3D imaging is preferred over 2D imaging, but requires a high imaging speed. Recent technical developments in imaging allow fast 3D reconstruction within seconds, but to capture fast movements associated with myofiber contraction (in particular of type 2X and 2B fibers), this is still not sufficiently fast.

Finite element modeling has revealed local strains and shear deformations within muscles being mechanically coupled by intra‐ and extra muscular connective tissue.^16^, ^24^ A combination of experimental data and mathematical modeling may provide valuable information to quantify the mechanical loads and cellular deformations that occur within the muscle tissue. Such knowledge can be used as input for studying effect of mechanical load (magnitude, frequency and loading type) on MuSC fate and function in vitro and ex vivo.

Overload‐induced hypertrophy and injury are accompanied by an increase in MuSC number. Activation and differentiation of MuSCs supports hypertrophy of myofibers,^49^ and therefore more knowledge of the existence of a mechanical loading threshold to induce an optimal response of MuSCs is crucial. The magnitude of mechanical loading, as well as frequency, duration and type (strain, pressure, or shear) might be determinants of this threshold. We hypothesize that MuSCs sense muscle force in a frequency and magnitude dependent manner.

Based on the above it is to be expected that, in sarcopenia, an impaired mechanosensitivity of aged MuSCs changes the mechanical loading threshold of MuSC activation. Currently, prevention or reversion of sarcopenia targets muscle protein synthesis, by means of nutritional supplementation either or not in combination with exercise training. The increased stiffness of the micro‐environment and the changes to the aged MuSC are underexposed in the context of mitigating sarcopenia. We propose that the mechanosensitivity of aged MuSCs is likely to be one of the determinants of impaired regeneration after injury and mechanical loading. Activation of aged MuSCs may require a different exercise protocol (e.g., duration, modality or intensity) compared to that of young MuSCs. In addition, restoration of mechanosensors in aged MuSC and subsequent transplantation into the muscle may be beneficial for reversion of muscle atrophy. Moreover, the increased stiffness of connective tissue of the ECM during aging is likely also affecting mechanosensitivity of MuSCs. Therefore, prevention or reversion of connective tissue stiffening will be required. This may be achieved, for example, by muscle stretching, muscle activation (eccentric or concentric), exercise (modality, duration and intensity), massage or nutritional interventions.

A combination of in vitro and in vivo studies is necessary to determine the effects of the abovementioned interventions on both niche stiffness and MuSC mechanosensitivity. Targeting both niche stiffness and MuSC mechanosensitivity is expected to result in improved muscle regeneration after injury and in response to mechanical loading.

## AUTHORS’ CONTRIBUTION

Heleen E. Boers (HEB), Mohammad Haroon (MH), Fabien Le Grand (FLG), Astrid D. Bakker (ADB), Jenneke Klein‐Nulend (JKN) and Richard T. Jaspers (RTJ) substantially contributed to drafting the paper or revising it critically. HEB, MH, FLG, ADB, JKN, and RTJ have read and approved the submitted and final version of this paper.
